# Case Report: Are We Witnessing an Increase of Chronic Ascending Aortic Dissection as a Collateral Effect to the COVID-19 Pandemic?

**DOI:** 10.3389/fcvm.2021.645135

**Published:** 2021-04-28

**Authors:** Arnaud Lyon, Ziyad Gunga, Lars Niclauss, Valentina Rancati, Piergiorgio Tozzi

**Affiliations:** ^1^School of Medicine, University of Lausanne, Lausanne, Switzerland; ^2^Service de Chirurgie Cardiaque, Centre Hospitalier Universitaire Vaudois, Lausanne, Switzerland; ^3^Département d'anesthésiologie, Hôpital Universitaire de Lausanne (CHUV), Lausanne, Switzerland

**Keywords:** ascending aorta, chronic aortic dissection, COVID−19, aortic surgery, delayed management

## Abstract

**Background:** The COVID-19 (coronavirus disease 2019) pandemic is reducing health care accessibility to non–life-threatening diseases, thus hiding their real incidence. Moreover, the incidence of potentially fatal conditions such as acute type A aortic dissection seems to have decreased since the pandemic began, whereas the number of cases of chronic ascending aortic dissections dramatically increased. We present two patients whose management has been affected by the exceptional sanitary situation we are dealing with.

**Case report:** A 70-year-old man with chest pain and an aortic regurgitation murmur had his cardiac workup delayed (4 months) because of sanitary restrictions. He was then diagnosed with chronic type A aortic dissection and underwent urgent replacement of ascending aorta and aortic root. The delay in surgical treatment made the intervention technically challenging because the ascending aorta grew up to 80 mm inducing strong adhesions and chronic inflammation. The second case report concerns a 68-year-old woman with right lower-limb pain who was diagnosed with deep vein thrombosis. However, a CT scan to exclude a pulmonary embolism could not be realized until 5 months later because of sanitary restrictions. When she eventually got the CT scan, it fortuitously showed a chronic dissection of the ascending aorta. She underwent urgent surgery, and the intervention was challenging because of adhesions and severe inflammation.

**Conclusion:** Delayed treatment due to sanitary restrictions related to COVID-19 pandemic is having a significant impact on the management of potentially life-threatening conditions including type A aortic dissection. We should remain careful to avoid COVID-19 also hitting patients who are not infected with the virus.

## Background

Acute Stanford type A aortic dissections (ATAADs) constitute critical emergencies that require immediate surgical treatment. This is due to the high risk of fatal complications, such as aortic rupture, severe aortic regurgitation, pericardial tamponade, and cerebral and coronary malperfusion (1), which are responsible for 33% mortality after 24 h and 50% mortality after 48 h (2). However, a very limited number of patients remain stable, with only mild to absent symptoms, and may thus survive the acute phase (1). After a 14-day period, the aortic dissection is defined as chronic (1). The global incidence of aortic dissection is 5 to 30 cases per 1 million people per year (3). We recently published our experience with ATAADs from 2014 to 2019. During the considered period, we treated 117 ATAADs in our center, which represents 3 to 5 ATAAD cases per month and had no chronic cases (4). However, between February and May 2020, the COVID-19 (coronavirus disease 2019) pandemic temporarily reduced health care accessibility. As such, these statistics were noticeably altered, with only three confirmed ATAADs, which represented a decrease of ¾, compared to the usual volume. Nevertheless, we experienced two cases of chronic type A aortic dissection (CTAAD) in July 2020, which is a pathology we usually see only once every 5 years (4).

This report illustrates the clinical implications of CTAAD that occurred in two patients shortly after the peak phase of the COVID-19 pandemic in our country.

## First Case Presentation

In February 2020, a 70-year-old man with a previously treated arterial hypertension consulted his general practitioner (GP) because of mild chest pain, without any other symptoms. The physical examination revealed a diastolic heart murmur that predominated in the aortic area. The patient's electrocardiogram (ECG) was normal, and the GP asked for cardiology advice. However, because of the sanitary restrictions due to the COVID-19 pandemic, the cardiologist examined the patient only 4 months later. The echocardiography revealed an aneurysm of the ascending aorta with signs of aortic dissection and severe aortic regurgitation.

**Figure 1:**
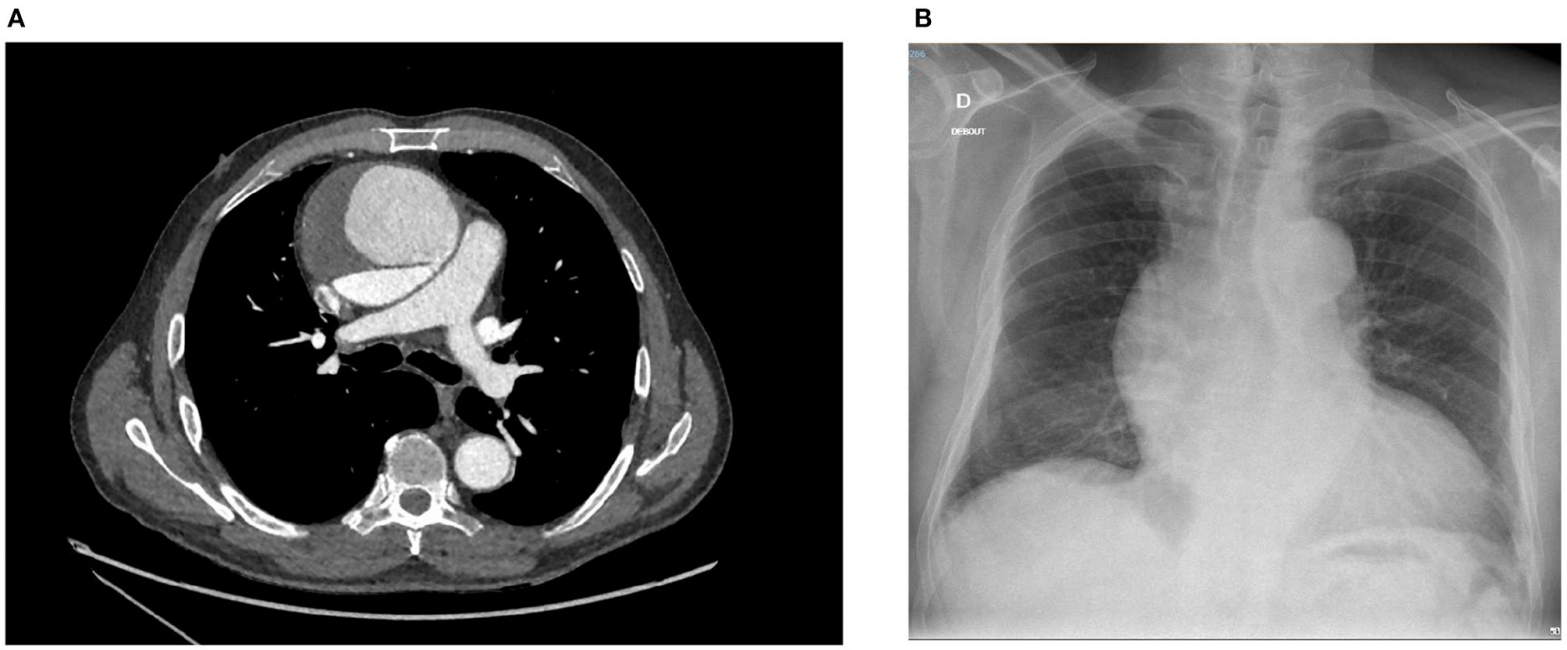
**(A)** Contrast medium thoracic CT scan showing a CTAAD with a thick intimal tear (7 mm) and an enlarged ascending aorta (82 × 87 mm). The false lumen is partially thrombosed. **(B)** Chest X-ray showing an aortic aneurysm with a widening of the mediastinal silhouette, an enlargement of the aortic knob, and a displacement of the trachea from the midline. CT, computed tomography; CTAAD, chronic type A aortic dissection.

A contrast medium thoracic computed tomography (CT) scan was immediately performed, which demonstrated an 82 × 87 mm aneurysm of the ascending aorta with a longitudinal tear of the intima that originated in a partially thrombosed circulating false lumen. The lesion began just above the ostium of the right coronary artery and extended up to the middle of the aortic arch with a 2.2-cm opening that was compatible with a Stanford type A aortic dissection. A 17-mm pericardial effusion was also identified (Figures 1A,B).

**Figure 2:**
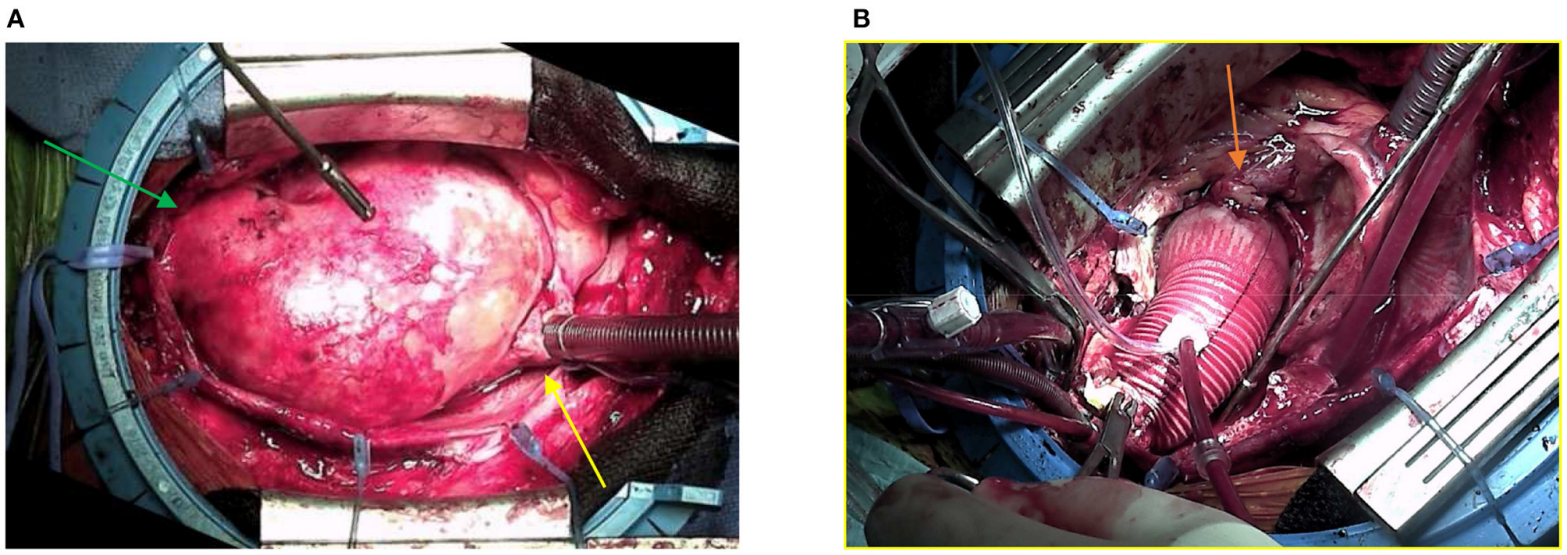
**(A)** Surgical view of the mediastinum after sternotomy. The huge aneurysm of the ascending aorta (82 × 87 mm) filled the whole cavity, displacing the heart into the left chest. The yellow arrow indicates the right atrium, and the green arrow indicates the aortic arch. **(B)** The ascending aorta has been replaced by the Dacron tube, leaving a free space that was previously filled by the aneurysm. The orange arrow indicates the reimplantation of the right coronary ostium.

**Figure 3:**
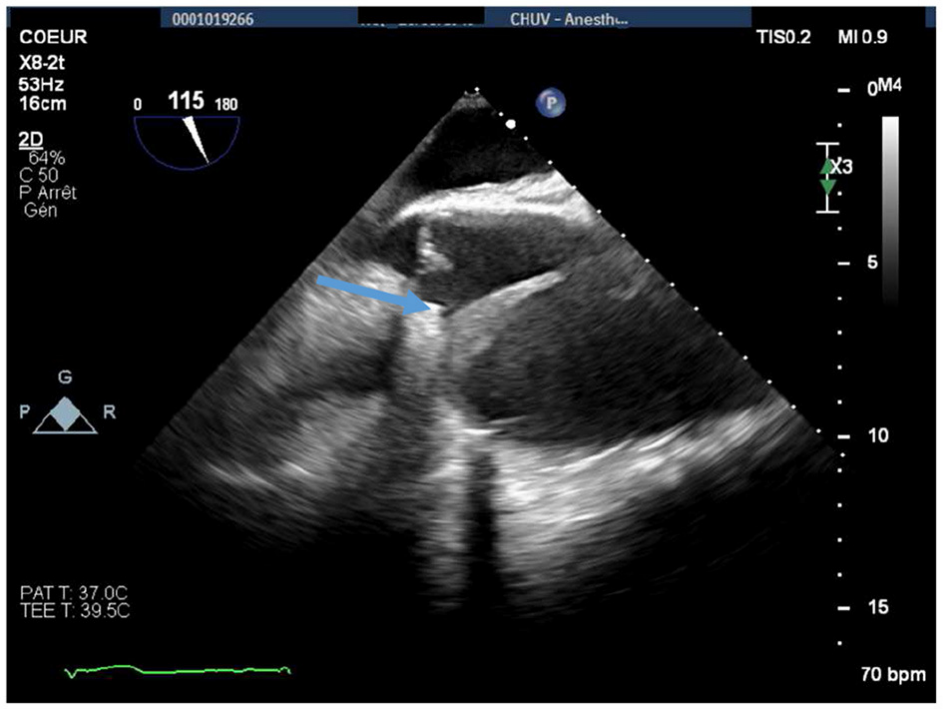
Perioperative midesophageal long-axis view showing a thickened (5 mm) dissection flap localized in the proximal ascending aorta, immediately after the origin of the right coronary artery (blue arrow). The maximal diameter of the ascending aorta was 74 mm.

The patient underwent replacement of the ascending aorta and aortic root (Bentall procedure with a 25-mm Carpentier–Edwards biological valve mounted on a Valsalva-type 28-mm Dacron tube). The intervention was technically difficult, as the heart was totally displaced into the left chest, and there were strong adhesions due to chronic inflammatory reactions (Figures 2A,B). The perioperative echocardiography showed a thickened dissection flap localized just above the origin of the right coronary artery (Figure 3). The right coronary reimplantation was therefore challenging because the ostium was fragilized by the dissection.

The post-operative period was uneventful, and the patient quickly recovered.

## Second Case Presentation

A 68-year-old female active smoker with known hypercholesterolemia, who had been treated for arterial hypertension and had a history of stroke in 2010 and pulmonary embolism in 2016, consulted her GP on February 25, 2020. She complained about the spontaneous onset of acute right lower-limb pain. The patient did not present any chest pain or dyspnea. The clinical examination revealed a painful but mild pretibial edema on the left lower limb, whereas no heart murmur was documented. d-Dimer levels were 570 mg/L (reference, <500 mg/L). On the same day, the patient was referred to the angiology department where a diagnosis of unprovoked deep vein thrombosis (DVT) of the left lower limb was established. Her arterial pressure was 131/86 mm Hg in the right arm and 134/87 mm Hg in the left arm, and all peripheral pulses were palpable. The patient was discharged with a therapeutic anticoagulation treatment (rivaroxaban 20 mg, once daily), with a 3-month follow-up examination scheduled for May. The follow-up found a favorable development, so the rivaroxaban was stopped and replaced by cardioprotective aspirin.

**Figure 4:**
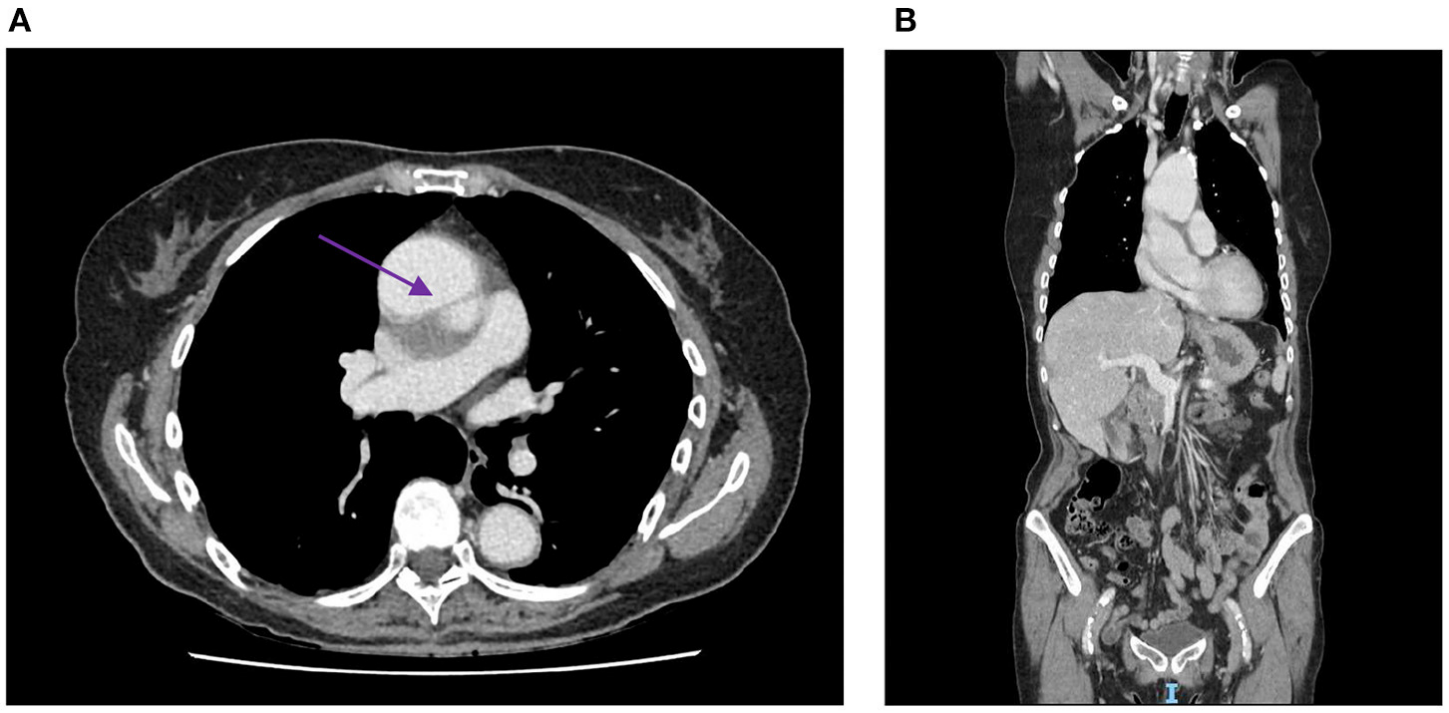
**(A)** Contrast medium CT scan showing an aneurysm of the ascending aorta (53 × 54 mm) with a tear of the intima, creating a thick false lumen (4 mm), which is indicated by the purple arrow. **(B)** Sagittal section of the contrast medium CT scan, which also shows the aneurysm and thick tear of the intima. CT, computed tomography.

In July, the patient's GP completed the diagnostic workup of her DVT with a CT scan. This examination unexpectedly showed an aneurysm of the ascending aorta (53 × 54 mm) with a chronic aortic dissection (Figures 4A,B). ECG was normal, and echocardiography found a ventricular function of 65% and a mild aortic insufficiency.

Based on the results of the CT scan, we performed an urgent repair of the dissected ascending aorta. We used a Gelweave 26-mm straight tube to replace the ascending aorta, just above the coronary ostia. The intervention was technically challenging, due to adhesions, but was ultimately uneventful, and the patient quickly recovered.

## Discussion

Delayed treatment of non–COVID-related diseases due to the COVID-19 pandemic is having a significant impact on patient safety even in developed countries such as Switzerland. Thousands of patients experience delayed management of potentially life-threatening conditions including type A aortic dissection. This is mainly due to a saturation of hospital capacity and patients' fear about becoming infected by the coronavirus in the hospital environment. We noticed a decrease in the number of ATAADs that were referred to our emergency department during the peak phase of the pandemic. This decrease of acute aortic syndromes was also highlighted by El-Hamamsy et al. who found the volume of ATAADs to be 76.5% lower than usual in New York City between March and April 2020 (5). Similar observations concerning myocardial infarctions and emergency department visits in general have also been reported (6, 7). Both of our patients exhibited atypical presentations with no specific symptoms or signs of ATAAD. We speculate that this played a role in their missed or delayed diagnoses. This may relate to the unexpected rise in CTAAD cases during the phase that followed the first peak of the pandemic. The absence of specific ATAAD symptoms in these patients was central to these delayed diagnoses. This phenomenon was reinforced by the pandemic, as patients altered their thresholds of symptoms that would normally compel them to seek medical advice. They waited longer before consulting a doctor than they would have before the pandemic.

## Conclusion

The apparent decrease in acute aortic dissections during the COVID-19 pandemic does not appear to be real, and it only relied on many patients not consulting, and remaining unnoticed, thus preventing them to get the medical care they deserved. Most of these patients probably passed away due to the complications of their aortic dissections, while the acute aortic dissections of those with mild or atypical symptoms who survived may have evolved into a chronic state that was only discovered when normal accessibility to health care services resumed.

The surgical treatment of CTAADs is more challenging with respect to acute dissection because it is associated to strong adhesions and consistent inflammatory reaction, significantly increasing the surgical mortality and morbidity. Therefore, delayed diagnosis also impacts the prognosis of patients with mild to absent symptoms (8).

The example of aortic dissections also illustrates the fact that patients affected by a wide range of diseases are directly impacted by the sanitary restrictions related to the COVID-19 pandemic. We thus conclude that more attention should be paid to avoid COVID-19 also hitting patients who are not infected with the virus.

### Limitations

Our case report is an observational study on a limited number of patients aimed at highlighting one of the possible consequences of the sanitary restrictions imposed by the authorities during the COVID-19 pandemic on the natural history of aortic dissection. By being a case report, it is not intended to clearly prove or bring statistical evidence of an association between the pandemic and an apparent increase of chronic aortic dissection cases. However, it shows a tendency in our center, which we believe is worth sharing with the medical community and which should be further investigated in a future larger epidemiological study.

## Data Availability Statement

The original contributions presented in the study are included in the article/supplementary material, further inquiries can be directed to the corresponding author/s.

## Ethics Statement

Written informed consent was obtained from the individual(s) for the publication of any potentially identifiable images or data included in this article.

## Author Contributions

AL is responsible for the literature research as well as the writing of the manuscript. PT contributed to this work as senior author. All authors contributed to the article and approved the submitted version.

## Conflict of Interest

The authors declare that the research was conducted in the absence of any commercial or financial relationships that could be construed as a potential conflict of interest.
